# Ascorbic acid for homogenous redox buffering in electrospray ionization–mass spectrometry

**DOI:** 10.1007/s00216-012-6196-z

**Published:** 2012-07-08

**Authors:** Sabine Plattner, Robert Erb, Jean-Pierre Chervet, Herbert Oberacher

**Affiliations:** 1 Institute of Legal Medicine, Innsbruck Medical University, Muellerstrasse 44, 6020 Innsbruck, Austria; 2 Antec, Industrieweg 12, 2382 NV Zoeterwoude, The Netherlands

**Keywords:** Mass spectrometry, Electrospray ionization, Electrochemistry, Redox buffering

## Abstract

Electrospray ionization (ESI) involves the dispersion of a liquid containing analytes of interest into a fine aerosol by applying a high potential difference to the sample solution with respect to a counter electrode. Thus, from the electrochemical point of view, the ESI source represents a two-electrode controlled-current electrochemical flow cell. The electroactive compounds part of the solvent sprayed may be altered by occurring electrolysis (oxidation in positive ion mode and reduction in negative ion mode). These reactions can be troublesome in the context of unknown identification and quantification. In the search for a simple, inexpensive, and efficient way to suppress electrochemical oxidation in positive ESI, the usability of ascorbic acid, hydroquinone, and glutathione for homogenous redox buffering was tested. Performance of the antioxidants was assessed by analyzing pharmaceutical compounds covering a broad range of functional groups prone to oxidation. Different emitter setups were applied for continuous infusion, flow injection, and liquid chromatography/mass spectrometry experiments. Best performance was obtained with ascorbic acid. In comparison to hydroquinone and glutathione, ascorbic acid offered superior antioxidant activity, a relatively inert oxidation product, and hardly any negative effect on the ionization efficiency of analytes. Furthermore, ascorbic acid suppressed the formation of sodiated forms and was able to induce charge state reduction. Only in the very special case of analyzing a compound isobaric to ascorbic acid, interference with the low-abundant [ascorbic acid+H]^+^ signal may become a point of attention.

**Electronic supplementary material:**

The online version of this article (doi:10.1007/s00216-012-6196-z) contains supplementary material, which is available to authorized users.

**Figure:**
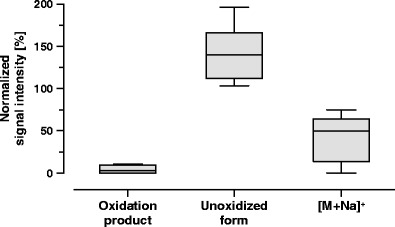
Ascorbic acid efficiently suppresses analyte oxidation and formation of sodiated forms in positive electrospray ionization

## Introduction

Electrospray ionization (ESI) is a soft ionization technique enabling the mass spectrometric analysis of polar and thermally labile compounds [1, 2]. ESI-mass spectrometry (MS) is particularly useful for the detection of small to very large (bio)organic molecules.

ESI involves the dispersion of a liquid containing analytes of interest into a fine aerosol. Formation of charged droplets is accomplished by applying a high potential difference to the sample solution with respect to a counter electrode. Hence, the ESI source can be viewed as a two-electrode controlled-current electrochemical flow cell [3–5]. In a typical setup used for ESI, an inert stainless steel capillary tube represents the working electrode and the sampling inlet of the mass spectrometer is the counter electrode.

ESI involves electrolysis (oxidation in positive ion mode and reduction in negative ion mode) of electroactive compounds part of the solvent sprayed. Electrochemical reactions may become of analytical importance if they change the composition of the sample solution. Of particular interest are processes that directly involve analytes. Typically, species with functional groups exhibiting low redox potentials are affected. In some cases redox processes are advantageous. They can, for instance, be used to create novel ionic species, to probe analyte (redox) chemistry, or to perform electrochemical ionization [6–16]. For any kind of analysis involving unknown compounds or quantification, however, electrochemical reactions are troublesome [17–19]. In this context, the ability to suppress analyte alteration would be an analytical advantage.

Parameters that determine the extent of occurring electrochemical reactions include the redox properties of components of the sample solution, mass transport characteristics as well as the interfacial potential and interfacial current at the emitter electrode. Limiting the mass transport to the emitter electrode is a means to suppress analyte electrolysis and can be accomplished for instance by increasing the solution flow rate, by reducing the electrode contact area, or by using a pulsed electrospray source [20, 21]. Another means of limiting analyte electrolysis makes use of controlling the interfacial potential. Direct and precise control of potential is possible by integrating the emitter as working electrode in a three-electrode emitter system [8, 22]. A more simple control over the interfacial potential can be accomplished by limiting the current available at the emitter electrode. Such control can be realized by implementing an upstream current loop [23, 24]. The simplest form of maintaining a particular interfacial potential employs redox buffering. Redox buffers, by undergoing electrolysis at the emitter electrode, “buffer” the potential to a level near the equilibrium potential for its reaction. Efficient redox buffers should possess relatively low oxidation potentials and neither the original compound nor its electrolysis product(s) should be detectable directly or indirectly by ESI-MS. Homogenous and heterogenous redox buffer systems have been presented, which included zinc capillary emitters [4], hydroquinone addition [25], copper capillary emitters [26], and polymer-modified emitter electrodes [27, 28]. Unfortunately, byproducts of some of these redox buffering approaches can appear in the mass spectra and/or they can chemically alter the analyte of interest [26, 27].

In search for a simple, inexpensive, and efficient way to suppress electrochemical oxidation in positive ESI, the usability of ascorbic acid, hydroquinone, and glutathione for homogenous redox buffering is assessed. These compounds represent important biological and industrial antioxidants. Pharmaceutical compounds covering a broad range of functional groups prone to oxidation are mixed with various amounts of the antioxidants and analyzed by ESI-MS. Performance in terms of antioxidant activity, reactivity of the oxidation product, and matrix effects (ion enhancement/suppression) is determined by continuous infusion, flow injection (FI), and liquid chromatography (LC)/MS experiments employing three different emitter systems for ionization to allow the selection of the most appropriate redox buffer.

## Materials and methods

### Chemicals

Acetonitrile, water, heptafluorobutyric acid (HFBA), acetic acid, l-ascorbic acid, hydroquinone, l-glutathione reduced, acetaminophen, amodiaquine dihydrochloride, doxepin hydrochloride, haloperidol, and diazepam were obtained from Sigma-Aldrich (St. Louis, MO, USA).

Morphine, methylendioxyamphetamine (MDA), and cocaine were purchased from Cerilliant (Round Rock, TX, USA).

Bunitrolol hydrochloride was obtained from Chemicals International (Holte, Denmark), zolpidem from Ratiopharm (Vienna, Austria), and nicotine from Merck Schuchardt (Hohenbrunn, Germany).

Norephedrine, levodopa, amphetamine, caffeine, olanzapine, imipramine, clomipramine, nalbuphine, octopamine, synephrine, aciclovir, sulfathiourea, reserpine, carbamazepine, diclofenac, ticlopidine, isoniazid, tamoxifen, trimethoprim, and sulfamethoxazole were part of our in-house collection of drug compounds.

Compounds analyzed by continuous infusion and FI-ES-MS are listed in Table 1. Samples were dissolved in 0.1 % acetic acid containing 50 % acetonitrile (*v*/*v*) and various amounts of ascorbic acid (0–570 μM), hydroquinone (0–910 μM), or glutathione (0–325 μM), respectively.

**Table 1:** Results of FI-ESI-MS experiments on the QqTOF instrument equipped with a planar electrode emitter

Compound	*c* (μM)	*E*_1/2_ (mV)	Oxidation product(s)	Relative peak area of oxidation product(s) (%)	Relative peak area of [M+Na]^+^ (%)
Imipramine	3.5	910	M−4	8	–
Tamoxifen	1.5	920	M+16 and M+14	<1, <1	–
Reserpine	1	970	M+16	2	–
Amodiaquine	5.5	1,030	M−2	6	–
Clomipramine	3	1,040	M−4	2	–
Morphine	11	1,060	M+16	21	–
Nalbuphine	5.5	1,200	M−2 and M+16	7, 13	–
Sulfathiourea	110	1,250	–		–
Olanzapine	16	1,330	M+16	2	–
Reproterol	5	1,430	–		–
Ticlopidine	4	1,460	–		–
Practolol	5	1,520	–		–
Acetaminophen	33	1,570	–		17
Sulfamethoxazole	40	1,610	–		53
Diclofenac	34	1,640	–		–
Levodopa	250	1,650	M−2	<1	–
Carbamazepine	8.5	1,660	–		62
Trimethoprim	3.5	1,670	–		–
Aciclovir	89	1,680	M+16	<1	7
Octopamine	98	1,860	–		–
Synephrine	30	1,880	–		–
Amphetamine	37	>2,000	–		–
Isoniazid	22	>2,000	–		–
Norephedrine	20	>2,000	–		–

“–” not detected

For LC/MS experiments, samples were dissolved in aqueous 0.02 % HFBA solution.

### Mass spectrometry

Mass spectrometric experiments were performed in positive ion mode on either a quadrupole–quadrupole–time-of-flight (QqTOF; QSTAR XL, AB Sciex, Foster City, CA, USA) or a quadrupole–quadrupole–linear ion trap mass spectrometer (QqLIT; QTrap 3200, AB Sciex).

**Fig. 1:**
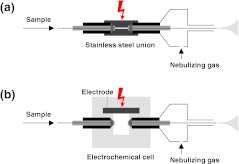
Schematic drawing of the ESI emitters used on the QqTOF instrument for **a** EC/MS as well as LC/MS experiments and **b** FI experiments

The QqTOF instrument was equipped with modified TurboIonSpray sources. Schematic drawings of the setups used are shown in Fig. 1. In one setup, the Peek tubing transfer line and the stainless steel sprayer capillary were replaced by fused silica capillaries (Fig. 1a; transfer line, 375 (o.d.) and 20 μm (i.d.); sprayer capillary, 90 (o.d.) and 20 μm (i.d.); Polymicro Technologies Phoenix, AZ, USA) [29, 30]. The spray voltage was applied to the stainless steel union. This type of sprayer was used for all LC/MS as well as electrochemistry (EC)/MS experiments on this instrument. In the second setup, the low-dead volume connection was replaced by a planar electrode device (Fig. 1b) provided by Antec (Zoeterwoude, The Netherlands) [20]. This device consisted of a cylindrical sandwich assembly containing a conductive diamond electrode (Magic Diamond, Antec) with an accessible area of approximately 15 mm^2^. The spray voltage was directly applied to the electrode. This type of sprayer was used for all FI experiments on the QqTOF mass spectrometer. Irrespectively of the sprayer design used, mass spectrometric parameters were optimized using a mixture of 500 ng/ml reserpine, 700 ng/ml amodiaquine, and 700 ng/ml amphetamine in 0.1 % acetic acid containing 50 % acetonitrile (*v*/*v*). Ion spray voltages ranged from 4 to 5 kV. Gas flows of 3 (nebulizing gas) and 10 (turbo gas) arbitrary units were employed. The temperature of the turbo gas was adjusted to 200 °C. The accumulation time was set to 1.0 s. Mass spectra were acquired from 100 to 700 and recorded on a personal computer with the Analyst QS software (version 1.0, service pack 8, AB Sciex).

The QqLIT instrument was equipped with a Turbo V source. The spray voltage (5 kV) was applied to a stainless steel capillary (200 mm × 100 μm i.d.). The integrated infusion pump was used to deliver samples at 15 μl/min for continuous infusion experiments. Mass spectrometric parameters were optimized using a mixture of 5 μg/ml reserpine, 2 μg/ml amodiaquine, and 5 μg/ml amphetamine in 0.1 % acetic acid containing 50 % acetonitrile (*v*/*v*). Gas flows of 30 (ion source gas 1) and 50 (ion source gas 2) arbitrary units were applied and the source temperature was set to 500 °C. Spectra were acquired in enhanced MS mode and recorded with Analyst 1.5 software (AB Sciex).

### Flow injection and liquid chromatography

FI and LC experiments were performed on a miniaturized ALEXYS HPLC system (Antec) online hyphenated to the QqTOF mass spectrometer [31–33]. The ALEXYS system was controlled by the Clarity software (DataApex, Prague, Czech Republic). The injection volume was 2 μl.

For FI experiments, the flow rate was 15 μl/min. A 0.1 % aqueous solution of acetic acid containing 50 % acetonitrile (*v*/*v*) was used as solvent.

Chromatographic columns with a length of 200 mm were prepared as described elsewhere [34, 35]. A retaining frit was made at one end of a polyimide coated fused-silica capillary tubing (200 μm, i.d.; Polymicro Technologies) by sintering a thin slug of 5.0 μm silica particles (Spherisorb S5W, Phase Sep, Queensferry, Clwyd, UK) wetted with a small droplet of sodium silicate solution (Sigma-Aldrich). Eurospher 100-5C18 particles (Knauer) were used as stationary phase. The flow rate was 3 μl/min. Chromatographic separations were accomplished with linear gradients of 5–95 % acetonitrile in aqueous 0.02 % HFBA solution containing 0–280 μM ascorbic acid within 10 min. The column temperature was held at 30 °C.

### EC-MS

The ROXY EC/LC system (Antec) was used to acquire mass voltammograms of the test compounds listed in Table 1 [31–33]. Electrochemical conversion was accomplished in an electrochemical thin layer cell (ReactorCell, Antec). The reactor cell consisted of a three electrode arrangement including a working electrode, counter electrode and a reference electrode. As working electrode a conductive diamond electrode (Magic Diamond, Antec) was used. The accessible area of the working electrode was 15 mm^2^. The inlet block of the cell was employed as counter electrode and the HyREF (H_2_/Pd; Antec) electrode was used as reference electrode. The working electrode and the counter electrode inlet block were separated by a 50-μm spacer giving a cell volume of approx. 750 nl. Potentials (0–2,000 mV) were applied using a purposive potentiostat (ROXY Potentiostat, Antec). The reactor cell was integrated into the autosampler system by placing it between the injection capillary and the injection valve. For electrochemical transformation, 1–250 μM sample solutions containing 0.1 % acetic acid and 50 % acetonitrile (*v*/*v*) were delivered through the electrochemical cell to the 2-μl injection loop at a flow rate of 4 μl/min. After injection, the sample solution was directly infused into the QqTOF mass spectrometer equipped with the sprayer shown in Fig. 1a. The flow rate was 15 μl/min. A 0.1 % aqueous solution of acetic acid containing 50 % acetonitrile (*v*/*v*) was used as solvent.

## Results and discussion

We have selected 24 compounds for studying the usefulness of ascorbic acid, hydroquinone, and glutathione as homogenous redox buffers (Table 1). Test compounds contained functional groups, which were expected to have rather low oxidation potentials. Functional groups covered included phenols, aliphatic amines, aromatic amines, heterocyclic amines, and sulfur-containing groups. The redox properties of the test compounds were assessed by EC/MS. The mass spectrometer was equipped with the emitter shown in Fig. 1a. Unwanted analyte oxidation during the ESI process was prevented by applying voltage to a low-dead volume stainless steel union. Samples were delivered by a FI system. Electrochemical oxidization was performed in an electrochemical cell integrated into the autosampler of the FI system. Electrochemical potentials of 0–2,000 mV were applied to a conductive diamond electrode. Compound-specific half-wave potentials (*E*_1/2_) were extracted from mass voltammograms obtained. *E*_1/2_ values ranged from 900 to 1,880 mV (Table 1). No oxidation was observed for amphetamine, isoniazid, and norephedrine on this particular electrode.

Next, the test compounds were analyzed by FI-ESI-MS. A planar electrode emitter was used to enhance analyte oxidation during the ESI process (Fig. 1b). The accessible area of the electrode was 15 mm^2^ ensuring efficient electrochemical oxidation at low flow rates (15 μl/min). Conductive diamond was employed as electrode material. Obtained experimental results are summarized in Table 1. Significant amounts of oxidation products were produced for imipramine, reserpine, amodiaquine, clomipramine, morphine, nalbuphine, and olanzapine. Dominant oxidation reactions included hydroxylation and dehydrogenation. Using the *E*_1/2_ values determined by EC/MS, an interfacial potential of approx. 1,000–1,250 mV was estimated.

In search for an efficient and inexpensive approach to suppress analyte oxidation in ESI, hydroquinone, ascorbic acid, and glutathione were tested as homogenous redox buffers. Glutathione and ascorbic acid are major antioxidants in biological systems. They are directly participating in the neutralization of free radicals and reactive oxygen species. Moreover, ascorbic acid is an important antioxidant additive to food and pharmaceutical products. Hydroquinone is an industrial antioxidant. It is commonly used as additive in photographic developers and as polymerization inhibitor. Furthermore, it was shown to represent a convenient redox buffer for ESI-MS [25, 27].

**Fig. 2:**
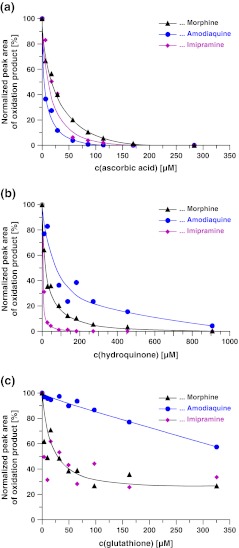
Evaluation of the antioxidant activity of **a** ascorbic acid, **b** hydroquinone, and **c** glutathione by FI-ESI-MS on the QqTOF instrument equipped with a planar electrode emitter using 21 pmol morphine, 7.0 pmol amodiaquine, or 11 pmol imipramine, respectively, as samples. The peak areas obtained for samples containing no antioxidant were used as reference points for normalization

The ability of various amounts of the antioxidants to suppress the electrochemical oxidation of morphine, amodiaquine, and imipramine was assessed by FI-ESI-MS with the planar electrode emitter. The obtained results are depicted in Fig. 2. The antioxidant activity induced by the addition of a certain amount of redox buffer to the sample solution was expressed by the relative change of the peak area of the oxidation product, and increased in the order glutathione < hydroquinone < ascorbic acid. Almost complete inhibition of oxidation was obtained with >175 μM ascorbic acid and >900 μM hydroquinone (Fig. 2a, b). Within the concentration range tested, glutathione provided only partial suppression of electrochemical oxidation (Fig. 2c).

Due to its comparatively low antioxidant activity and its tendency to ionize in positive ion mode giving rise to ion suppression, glutathione was ruled out as appropriate redox buffer for ESI in positive ion mode.

**Fig. 3:**
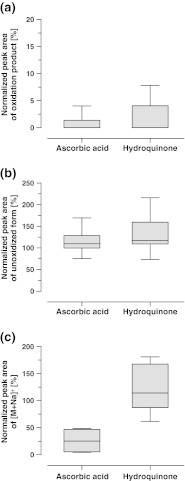
Impact of the addition of the antioxidants ascorbic acid (570 μM) and hydroquinone (910 μM) on normalized peak areas of **a** the oxidation products and **b**, **c** the unoxidized forms ([M+H]^+^ and [M+Na]^+^) obtained by FI-ESI-MS on the QqTOF instrument equipped with a planar electrode emitter. Samples, **a** morphine, olanzapine, imipramine, clomipramine, nalbuphine, reserpine, and amodiaquine; **b** all 24 test compounds; **c** acetaminophen, aciclovir, carbamazepine, and sulfamethoxazole. The peak areas obtained for samples containing no antioxidant were used as reference points for normalization

Antioxidant activities of ascorbic acid (570 μM) and hydroquinone (910 μM) that were obtained for seven test compounds oxidized in the planar electrode emitter are summarized in Fig. 3a. Both redox buffers efficiently suppressed oxidation. For sample solutions containing an antioxidant, peak areas of the oxidation products were <8 % of the peak areas observed for crude sample solutions.

Ascorbic acid and hydroquinone seem to have a positive effect on ionization efficiency. Average normalized peak areas increased by 14 ± 25 % for ascorbic acid and by 28 ± 35 % for hydroquinone, respectively.

Within the mass spectra of four compounds (acetaminophen, sulfamethoxazole, carbamazepine, and aciclovir) signals corresponding to sodiated forms were observed (Table 1). The impact of the redox buffer addition on the peak areas of these species is depicted in Fig. 3c. Ascorbic acid seems to suppress adduct formation; it induced a more than 50 % reduction of the peak areas corresponding to the sodiated forms. Contrarily, hydroquinone seems to have a promotive effect on adduct formation; on average peak areas increased by 17 %.

Hydroquinone seems to efficiently suppress analyte oxidation in ESI. This compound, however, was ruled out to represent a convenient redox buffer due to its promoting effect on adduct formation and due to the known tendency of its oxidation product 1,4-benzoquinone to react with thiol functional groups [13, 27, 36].

Experiments with the planar electrode emitter revealed that ascorbic acid represents an appropriate redox buffer. Ascorbic acid efficiently suppresses electrochemical oxidation by modulating the interfacial potential. Furthermore, ascorbic acid was found to inhibit the formation of sodium adducts. Thus, the addition of ascorbic acid has a positive effect on mass spectrometric detection sensitivity and reduces spectral complexity. A clear advantage of ascorbic acid over hydroquinone is the relatively low reactivity of its oxidized form dehydroascorbic acid. Although it has been reported that dehydroascorbic acid and its degradation products might react with amines, amino acids or proteins in Maillard-type chemistry [37, 38], in our case none of the test compounds was altered. This observation suggests that reactions with dehydroascorbic acid are unlikely to occur.

In a further set of experiments, the usability of ascorbic acid as redox buffer on a more traditional emitter system was evaluated. The experiments were performed on the QqLIT system employing a commercially available sprayer setup (Turbo V, AB Sciex). In the sprayer system tested the voltage was applied to a stainless steel capillary (200 mm × 100 μm i.d.). Samples were delivered by continuous infusion. The flow rate was set to 15 μl/min to allow rather efficient electrochemical oxidation. Observed oxidation products are summarized in Table S1 in the Electronic supplementary material (ESM). Significant amounts of oxidation products were produced for reserpine, amodiaquine, sulfathiourea, and olanzapine. Dominant oxidation reactions included hydroxylation and dehydrogenation.

The impact of ascorbic acid addition (570 μM) on mass spectrometric detection with the stainless steel capillary emitter is shown in Fig. S1 in the ESM. Redox buffering led to a significant decrease of the signal intensities of the oxidation products; the reduction of the signal intensity was in all cases >85 %. Furthermore, ascorbic acid gave rise to signal enhancement and adduct suppression. The average increase of signal intensity of the unoxidized form was 41 %; the average reduction of the signal intensity of the sodiated form was 57 %.

Another effect on ESI-MS of small (bio)organic molecules induced by ascorbic acid is shown in Fig. S2 in the ESM. For multiply charged ions, ascorbic acid may give rise to charge state reduction [39]. This effect was exemplified for olanzapine. Most probably as the result of occurring proton transfer reactions, increasing amounts of ascorbic acid slightly shifted peak intensity from the +2 charge state to the +1 charge state. This observation was consistent with the effect of ascorbic acid on the charge state distribution of proteins in ESI-MS [40, 41].

ESI is an important interface to hyphenate LC with MS. For LC/MS applications, the solvent system cannot be optimized independently for one technique, neither for LC nor for ESI-MS. Retention characteristics and ionization efficiencies are strongly determined by eluent properties. In general, ESI is most efficient with solutions having low viscosity, low surface tension, high volatility, and low ionic strength. Such conditions, however, can negatively affect chromatographic separation. Usually, a compromise has to be found to enable the efficient on-line coupling of both techniques [35].

**Fig. 4:**
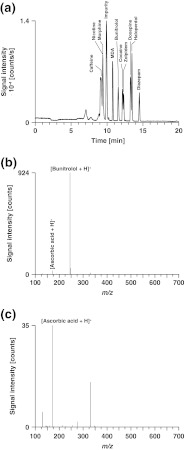
LC/MS analysis of a mixture of ten test compounds. **a** Total ion current chromatogram, **b** mass spectrum of bunitrolol extracted at 11.6 min, **c** mass spectrum of the background extracted at 11.2 min. Column, Si-C18, 5 μm, 200 × 0.20 mm i.d.; mobile phase, water containing 0.02 % aqueous HFBA and 280 μM ascorbic acid (A), acetonitrile containing 0.02 % HFBA and 280 μM ascorbic acid (B); linear gradient, 5–95 % B in 10 min; flow rate, 3 μl/min; temperature, 30 °C; injection volume, 2.0 μl; sample, 10.2 pmol caffeine, 3.6 pmol nicotine, 10.4 pmol morphine, 9.2 pmol MDA, 1.6 pmol bunitrolol, 2.0 pmol cocaine, 2.0 pmol zolpidem, 0.8 pmol doxepin, 0.6 pmol haloperidol, and 0.8 pmol diazepam

The effect of various concentrations of ascorbic acid (0–280 μM) on the reversed-phase LC separation of a mixture of pharmaceutical compounds can be deduced from Fig. S3 in the ESM. Chromatographic separations were accomplished on a Si-C18 column by gradients of acetonitrile in 0.02 % HFBA containing different amounts of ascorbic acid as redox buffer. ESI-MS was performed on the QqTOF instrument equipped with the low-dead volume emitter (Fig. 1a). The addition of ascorbic acid to the mobile phase had hardly any impact on chromatographic separation. Retention times (*t*_r_) and peak widths at half height (*w*_1/2_) remained almost constant (see Fig. S3a, b in the ESM). A representative total ion current chromatogram is depicted in Fig. 4a. The mobile phase contained 280 μM ascorbic acid. The mass spectrum of bunitrolol is shown in Fig. 4b. Besides the protonated form of bunitrolol, a signal corresponding to the protonated form of ascorbic acid was detected. However, the [ascorbic acid+H]^+^ intensity was low even at very high ascorbic acid concentrations (Fig. 4c). Of particular importance is the fact that within the concentration range tested hardly any alteration of analyte signal intensities was observed (see Fig. S3c in the ESM), which clearly suggests that ion suppression induced by ascorbic acid can be neglected.

The LC/MS experiments revealed that ascorbic acid is a convenient mobile phase additive. At concentrations sufficiently high to suppress analyte oxidation (>175 μM), neither chromatographic separation nor mass spectrometric detection is affected. Only in the very special case of analyzing a compound isobaric to ascorbic acid, interference with the [ascorbic acid+H]^+^ signal may become a point of attention.

## Conclusions

Homogenous redox buffering with ascorbic acid represents a simple, efficient, and inexpensive means of controlling analyte oxidation in positive ion mode ESI. Ascorbic acid can be used as solvent additive for continuous infusion, FI and LC/MS experiments. In comparison to hydroquinone and glutathione, ascorbic acid offers superior antioxidant activity, a relatively inert oxidation product, and low ionization efficiency. Furthermore, ascorbic acid suppresses the formation of sodiated forms and is able to induce charge state reduction. Only in the very special case of analyzing a compound isobaric to ascorbic acid, the interference with the low-abundant [ascorbic acid+H]^+^ signal has to be considered.

Our study focused on the oxidation of pharmaceutical compounds. A wide range of different functional groups prone to oxidation during ESI were covered. Therefore, we believe that the homogenous redox buffer ascorbic acid might be applicable to other types of analytes as well. It could be particularly beneficial for the analysis of peptides and proteins.

Antioxidants are very important ingredients in dietary supplements, pharmaceuticals and cosmetics. In search for new, more efficient additives, the determination of the antioxidant activity of a compound is of utmost importance. We have successfully applied a FI-ESI-MS setup employing a planar electrode emitter for analyte oxidation for comparing the antioxidant activities of ascorbic acid, hydroquinone and glutathione. Therefore, we believe that electrochemical devices hyphenated to MS have the capability to complement existing assays for studying antioxidative properties.

## Electronic supplementary material

Below is the link to the electronic supplementary material.

ESM 1 (PDF 39.5 kb)
