# Supplementary material for: Ascorbic acid for homogenous redox buffering in electrospray ionization–mass spectrometry
Source: Anal Bioanal Chem. 2012 Jul 8;404(5):1571–9. doi: 10.1007/s00216-012-6196-z (PMC3426670; doi:10.1007/s00216-012-6196-z)

Analytical and Bioanalytical Chemistry

Electronic Supplementary Material

Ascorbic acid for homogenous redox buffering in electrospray ionization–mass spectrometry

Sabine Plattner, Robert Erb, Jean-Pierre Chervet, Herbert Oberacher

Table S1. Compounds used for studying the impact of ascorbic acid on mass spectrometric detection by continuous infusion ESI-MS on the QqLIT instrument equipped with a stainless steel capillary emitter.

Compound	c [µM]	Oxidation product(s)	Relative peak area of oxidation product(s) [%]	Relative peak area of $[M+Na]^+$ [%]
Tamoxifen	3.0	M+16	<1	2
Reserpine	8.0	M-2, M+16	10, 6	<1
Amodiaquine	5.5	M-2	8	-
Clomipramine	16	M-2	<1	-
Nalbuphine	14	M+16	<1	<1
Sulfathiourea	43	M-2	2	4
Olanzapine	16	M-2	12	-
Reproterol	13	-		11
Practolol	4	M-2	<1	5
Acetaminophen	66	-		24
Sulfamethoxazole	79	-		53
Diclofenac	65	-		295
Carbamazepine	8.5	-		156
Aciclovir	44	-		12

-, not detected

Figure S1. Impact of the addition of the antioxidant ascorbic acid (570 µM) on the normalized signal intensities of the oxidation products and the unoxidized forms ($[M+H]^+$, $[M+Na]^+$) obtained by continuous infusion ESI-MS on the QqLIT instrument equipped with a stainless steel capillary emitter. Samples, (a) olanzapine, sulfathiourea, reserpine, amodiaquine; (b) all 14 test compounds; (c) acetaminophen, nalbuphine, practolol, reproterol, aciclovir, sulfathiourea, carbamazepine, diclofenac, tamoxifen, sulfamethoxazole. The peak areas obtained for samples containing no antioxidant were used as reference points for normalization.

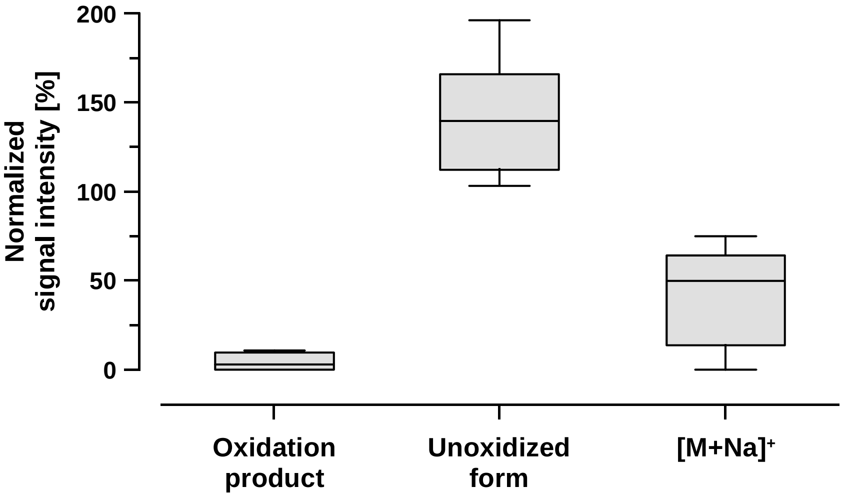

Figure S2. Charge state reduction for olanzapine induced by the addition of 0-280 µM ascorbic acid to a 16 µM solution of the test compound analyzed by continuous infusion ESI-MS on the QqLIT instrument equipped with a stainless steel capillary emitter.

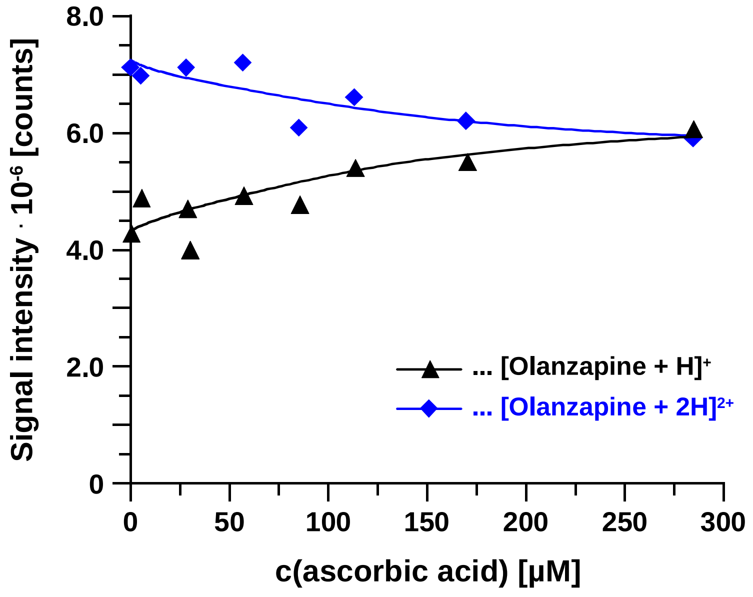

Figure S3. Impact of the addition of ascorbic acid as homogenous redox buffer on (a) normalized retention times, (b) normalized peak widths at half height, and (c) normalized signal intensities for 10 test compounds analysed by LC/MS. Column, Si-C18, 5 µm, 200 x 0.20 mm i.d.; mobile phases, (A) water containing 0.02% aqueous HFBA and 0-280 µM ascorbic acid, (B) acetonitrile containing 0.02% HFBA and 0-280 µM ascorbic acid; linear gradient, 5-95% B in 10 min; flow rate, 3 µl/min; temperature, 30 °C; injection volume, 2.0 µl; sample, 10.2 pmol caffeine, 3.6 pmol nicotine, 10.4 pmol morphine, 9.2 pmol MDA, 1.6 pmol bunitrolol, 2.0 pmol cocaine, 2.0 pmol zolpidem, 0.8 pmol doxepin, 0.6 pmol haloperidol and 0.8 pmol diazepam. The peak areas obtained for samples containing no antioxidant were used as reference points for normalization.

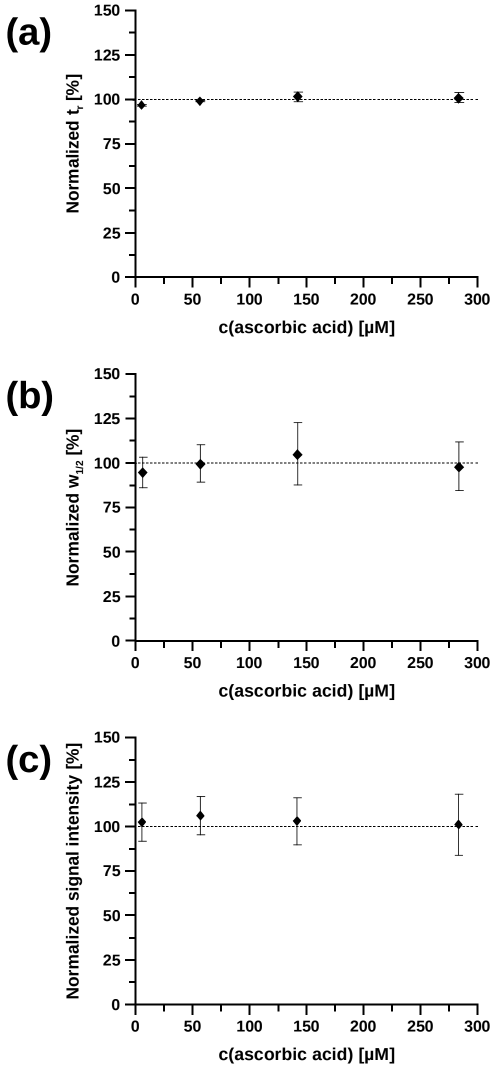

Supplement: Supplementary file 1 — (PDF 39.5 kb) [file 216_2012_6196_MOESM1_ESM.pdf]
